# Putative Drone Copulation Factors Regulating Honey Bee (*Apis mellifera*) Queen Reproduction and Health: A Review

**DOI:** 10.3390/insects10010008

**Published:** 2019-01-08

**Authors:** Laura M. Brutscher, Boris Baer, Elina L. Niño

**Affiliations:** 1 Department of Entomology and Nematology, University of California, Davis, CA 95616, USA; 2 Centre for Integrative Bee Research, Department of Entomology, University of California, Riverside, CA 92521, USA; boris.bar@ucr.edu

**Keywords:** honey bees, reproduction, queens, drones, seminal fluid, pathogens

## Abstract

Honey bees are major pollinators of agricultural and non-agricultural landscapes. In recent years, honey bee colonies have exhibited high annual losses and commercial beekeepers frequently report poor queen quality and queen failure as the primary causes. Honey bee colonies are highly vulnerable to compromised queen fertility, as each hive is headed by one reproductive queen. Queens mate with multiple drones (male bees) during a single mating period early in life in which they obtain enough spermatozoa to fertilize their eggs for the rest of their reproductive life span. The process of mating initiates numerous behavioral, physiological, and molecular changes that shape the fertility of the queen and her influence on the colony. For example, receipt of drone semen can modulate queen ovary activation, pheromone production, and subsequent worker retinue behavior. In addition, seminal fluid is a major component of semen that is primarily derived from drone accessory glands. It also contains a complex mixture of proteins such as proteases, antioxidants, and antimicrobial proteins. Seminal fluid proteins are essential for inducing post-mating changes in other insects such as *Drosophila* and thus they may also impact honey bee queen fertility and health. However, the specific molecules in semen and seminal fluid that initiate post-mating changes in queens are still unidentified. Herein, we summarize the mating biology of honey bees, the changes queens undergo during and after copulation, and the role of drone semen and seminal fluid in post-mating changes in queens. We then review the effects of seminal fluid proteins in insect reproduction and potential roles for honey bee drone seminal fluid proteins in queen reproduction and health. We finish by proposing future avenues of research. Further elucidating the role of drone fertility in queen reproductive health may contribute towards reducing colony losses and advancing honey bee stock development.

## 1. Introduction/Background

Insect pollination of commercial crops is valued worldwide at $175 billion annually and pollination services provided by commercially managed honey bee (*Apis mellifera*) colonies in the United States alone are valued at $14.6 billion annually [1]. However, commercial beekeepers in the US have reported up to 45% annual colony losses since 2006 [2,3,4,5,6,7,8,9]. Multiple factors have been implicated, including agrochemical exposure, forage quality and availability, management practices, parasites, pathogens, and queen reproductive failure [2,3,4,5,6,7,8,9]. However, factors specifically impacting queen fertility and their subsequent roles in colony health have received limited attention. In fact, commercial beekeepers have frequently reported queen failure and the ectoparasitic mite *Varroa destructor* as the two most common reasons for colony losses in the past several years [4,10]. Queen failure occurs when the queen is no longer reproductively fit and she stops efficiently laying eggs or begins laying unfertilized eggs that become drones (male bees) [11]. While queens can typically live 2–3 years, commercial beekeepers have started replacing queens at least once per year due to poor queen quality and frequent queen failure [12].

Honey bee colonies are composed of tens of thousands of sterile female workers, hundreds to thousands of seasonal haploid male drones, and a single queen, the only member of the colony that can lay both fertilized and unfertilized eggs [13]. Roughly a week after emerging, virgin queens undertake one to a few nuptial flights over several days [13]. Honey bee queens are polyandrous and they mate with multiple drones, which reach sexual maturity about two weeks after emergence [13,14,15,16,17,18,19,20]. During a nuptial flight, the queen flies up to 3 km away from her hive to rendezvous with thousands of drones at a drone congregation area (DCA), located 5–40 m above ground [20]. Older reports have determined that queens mate with an average of 12 drones [13,21], but recent work found that queens can mate up to 34–77 drones [22]. During copulation, the drone irreversibly everts its endophallus into the female, transfers his semen into the oviduct, and drops to the ground to die [20,23]. Roughly 10% of each male’s ejaculate is transferred into the queen’s oviduct [13,20,24,25]. 

Once a queen has terminated her final nuptial flight and returns to the hive, she starts to store sperm in her spermatheca, a specialized organ found in many insects to facilitate spermatozoa storage, and commences egg laying [20]. Only about 3% to 5% of ejaculated spermatozoa from each drone is stored in the queen’s spermatheca for future egg fertilization [13,20,24,25]. A queen can store approximately five to six million total spermatozoa in her spermatheca [14,20,26]. While it varies based on number of stored spermatozoa, queens are highly efficient and fertilize each egg with a median of two spermatozoa; queens that are inseminated with more semen tend to store more spermatozoa and, in turn, fertilize eggs with more spermatozoa [27]. Honey bees are parthenogenic and queens lay both fertilized eggs that hatch into diploid female workers or queens and unfertilized eggs that develop into haploid drones [20]. The type of egg that is laid depends on the type of comb cells into which the queen is laying—larger cells are reserved for drones while worker eggs are laid into smaller cells [20]. In addition to her role as the primary reproductive female in a colony, the queen also continuously releases a blend of pheromones that passively maintain social cohesion of the hive and other aspects of colony organization [13]. More comprehensive information on the mating biology of honey bees can be found in Reference [20].

Honey bee queens are typically assessed for their quality based on reproductive longevity, potential amount of viable brood they can produce, the number of the drones with which they have mated, and the genetic diversity of her mates [28]. There are several traits that are associated with queen quality, including overall weight [11,28,29,30,31,32,33], weight of the ovaries [34,35,36], weight of the spermatheca, and the number of viable stored spermatozoa [11,28,29,37,38,39,40]. Queen quality is impacted by the age at which larvae are nutritionally directed into the queen developmental pathway via continued feeding of royal jelly [12,41,42,43]. Ideally 1st instar larvae are used, but older larvae may be reared into queens if the mother queen is unexpectedly lost [12,41,42,43]. Queen quality is also affected by genetic background [29], pesticide exposure [44,45,46], and parasite or pathogen load [28]. For a more comprehensive review regarding the relationship of these factors to queen quality, see Reference [28].

Importantly, queen reproduction is also affected by mating conditions [47,48,49,50,51,52,53]. When a queen mates with drones, she undergoes extensive behavioral, physiological, and molecular changes, including reduced sexual receptivity, ovary development, ovulation, modulation in pheromone production, and transcriptional regulation. These changes contribute to aspects of queen reproductive quality with potential far-reaching implications [47,54,55,56,57,58]. Studies utilizing instrumental insemination have determined that drone semen and seminal fluid, a major component of semen containing numerous proteins and metabolites, initiates many of these post-mating changes in queens and likely plays an important role in shaping queen quality [47,48,49,50,51,52,53]. However, the specific molecules in semen and seminal fluid involved in initiating post-mating changes in queens have yet to be identified. Hereinto, we review the currently available work investigating post-mating changes in queens and the different copulation factors that influence queen fertility. We then cover recent work identifying the proteins in drone seminal fluid and their potential roles in queen quality and post-mating changes. Furthermore, since queens are polyandrous, they are at greater risk of being infected with sexually transmitted pathogens, such as *Nosema* spp. or Deformed Wing virus, which may threaten their health and fitness [59,60,61,62]. Therefore, we also review research investigating diseases and antimicrobial mechanisms of drone seminal fluid and its ability to reduce parasite transmission during mating. We conclude by discussing future avenues of research.

## 2. Honey Bee Queen Post-Mating Changes

**Figure 1:**
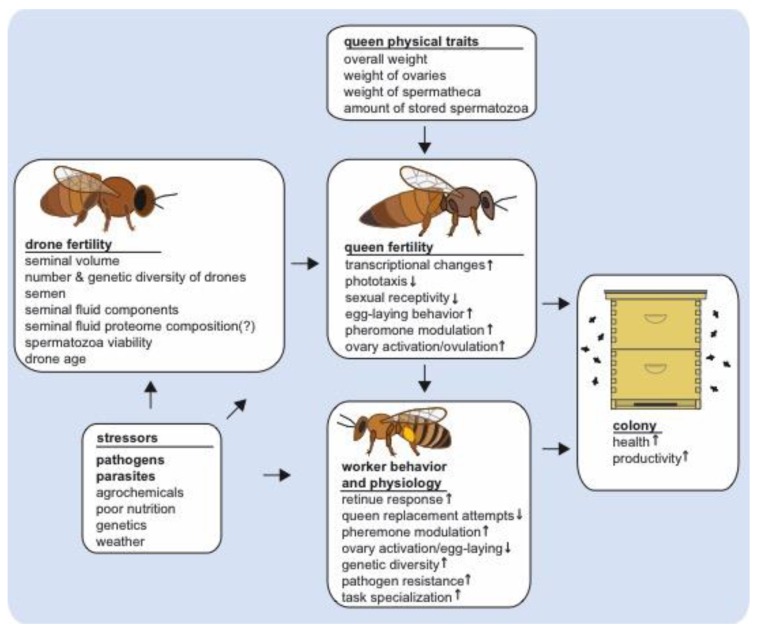
Stressors impacting queen and drone fertility and their downstream effects on queen quality and post-mating changes and worker behavior and physiology. The small arrows inside of the boxes indicate an increase or decrease of queen post-mating changes or subsequent worker behavioral or physiological traits as observed during ideal natural conditions, but disruptions in male fertility may subsequently affect the intensity and manifestation of these phenotypes. Multiple stressors impact queen and drone fertility, including pathogens and parasites, such as *Nosema* spp. and *Varroa destructor* mites, which affect drone mating flight behavior and seminal fluid proteome composition. These and other drone mating factors, such as insemination volume and insemination fluid composition also impact queen fertility, which subsequently affect the behavior and physiology of workers. In turn, altered queen-worker interactions may affect colony level productivity and health.

Mating only occurs for a short period early in a queen’s life, but it initiates multiple post-mating changes that impact queen reproduction and potentially colony health and productivity (Figure 1) [47,49,54,55,56,57].

### 2.1. Behavioral Post-Mating Changes 

Virgin queens are highly phototatic (attracted to light) and undertake one or more nuptial flights before they cease performing mating flights [13]. Once the queen has taken her final mating flight and has stored spermatozoa in her spermatheca, she permanently exhibits reduced phototaxis and sexual receptivity. The queen remains in the hive to lay eggs, unless she participates in a swarming event [13,63]. Swarming is initiated by the production of virgin queens, followed by the departure of the resident queen with approximately half of the workers [13]. The remaining workers then attend the new virgin queens [13]. Post-mating changes in the queen’s pheromones, a crucial mode of chemical communication between honey bees, also alter the behavior of surrounding workers in the hive [47,48,49,51,54,55,64,65]. For example, mated queens and older virgins are more readily accepted and elicit a greater worker retinue response than virgin queens because older virgins and mated queens produce a more complete suite of pheromones [47,55,66,67]. The retinue response is defined by workers licking and antennating the queen to transmit her pheromones throughout the colony [13]. Aged virgins eventually exhibit ovary activation and increased pheromone production, but mated queens still produce more pheromones and are more attractive to workers than older virgins [55,68]. In addition, if the workers are acclimated to the pheromone profile of one queen, they may aggressively respond to queens with “foreign” pheromone profiles by surrounding and balling them [69]. Pheromones produced from mated queens also inhibit ovary activation, egg-laying, and queen cell building and rearing in workers [47,48,49,51,54,55,64,65]. Furthermore, the colonies headed by mated queens collect more pollen then colonies headed by virgin queens [70]. This has substantial consequences for long term colony survival because pollen collection is associated with winter survival [71].

### 2.2. Physiological Post-Mating Changes

The ovaries of queens are essential for egg production and are approximately eight times bigger in mated, egg-laying queens compared to virgin queens [72,73]. Mating also has a strong impact on queen pheromone production [47,48,49,50,51,52,53]. The queen possesses several glands (i.e., mandibular, labial, Dufour’s, tergal, and tarsal) that produce pheromones that are essential for maintaining colony social organization and are substantially different from those of workers or males [69,74,75,76,77,78,79,80,81,82,83].

The best-studied glands are the queen’s mandibular glands which are large sacs attached to the mandibles and generate queen mandibular pheromone (QMP), which is primarily composed of five chemicals: (*E*)-9-keto-2-decenoic acid (9ODA), (*R*,*E*)-(−)- and (*S*,*E*)-(+)-9-hydroxy-2-decenoic acid (9HDA), methyl p-hydroxybenzoate (HOB), and 4-hydroxy-3-methoxyphenylethanol (HVA) [47,48,49,50,51,52,54,74,77]. Pheromones produced in the queen’s mandibular gland are largely responsible for inducing the behaviors that other castes exhibit when headed by a mated queen: Worker retinue response, reduced rearing of new queens, and reduced swarming [69,75,77,78,79,80,81,82,83]. It also slows the ontogeny of foraging and regulates pollen collecting behavior [69,75,77,78,79,80,81,82,83]. In addition to having effects on worker behavior, QMP also inhibits worker ovary activation, increases worker resistance to starvation, and induces changes in worker brain and fat body gene expression [69,75,77,78,79,80,81,82,83]. Virgin queens, six and 12 days after eclosion, exhibit low levels of HOB and moderate levels of 9HDA and 9ODA in their mandibular glands, whereas one-year-old mated queens exhibit increased levels of HOB, HVA, 9ODA, and 9HDA [54,55,84]. Interestingly, the mandibular pheromone of queens that are newly mated (two days post-natural mating) are more similar to that of virgin queens than queens one year after mating [49]. Thus, pheromone production does not immediately change after mating [49].

The Dufour’s gland also elicits retinue responses in workers and may signal ovary activation when produced in workers [85,86]. The Dufour’s gland is located near the dorsal vaginal wall and its secretions are primarily composed of hydrocarbons (e.g., tricosane, pentacosanes, heptacosane) and esters [65]. Mated queens typically produce greater relative amounts of hydrocarbons and lower relative amounts of esters in the Dufour’s gland as compared to unmated queens [65]. The tergal glands also elicit the retinue response, but they do not evoke as strong of a response compared to QMP [87]. After natural mating, tergal glands produce greater amounts of alkenes [88]. Less is known about the composition and function of other pheromone-producing glands in queens.

### 2.3. Molecular Post-Mating Changes

After copulation, queens exhibit transcriptional and protein-level changes in the spermatheca, ovaries, and brain [57,68,89,90,91]. Specifically, mated queens exhibit higher expression of antioxidant genes in the spermatheca, which may aid in maintaining the viability of stored spermatozoa [91]. Previous studies have utilized microarray analyses to determine what genes are differentially regulated in the brains and ovaries of virgin queens, recently-mated queens, and egg-laying queens (Table 1) [57,68]. Ovary activation, ovary gene expression, and mandibular gland pheromone composition are correlated with each other, indicating that these processes are linked temporally and/or mechanistically [57,68]. Likewise, the pheromone composition and ovary gene expression profiles of recently-mated queens are more similar to virgin queens than they are to laying queens [57,68].

In addition, brain gene expression and sexual receptivity are correlated [57,68]. Interestingly, natural mating induces differential expression of vision, chemo-reception, metabolic, and immune-related genes as compared to virgins, which may be linked with reduced queen phototaxis and sexual receptivity after mating [92]. Similarly, carbon dioxide (CO_2_), a substance commonly used to sedate queens during instrumental insemination and also happens to induce queen ovary activation, causes effects similar to mating by inducing changes in gene expression in the brains of queens, such as reduced expression of cognition and vision-related genes [92,93]. Reproductively activated bumble bee queens (*Bombus terrestris*) and distantly related *Drosophila melanogaster* regulate expression of vision-related genes involved in phototransduction after mating [94,95]. Thus, these processes are likely to be conserved across insects.

Mating also alters the morphology of the brain [90] and results in differential protein levels of neurotransmitters and their associated metabolites in the brain [89]. These changes may also be related to the behavioral changes and ovary activation observed in queens. Specifically, mated queens exhibit lower levels of the biogenic amine dopamine as compared to virgin queens [89]. Similarly, when exposed to CO_2,_ queens exhibit decreased brain gene expression of dopamine receptors and activation of ovaries [96]. In contrast, workers also produce less dopamine in the brain, but ovary activation is inhibited after CO_2_ exposure [97]. This suggests that dopamine plays important and contrasting roles in regulating ovary activation in honey bees [96]. 

In summary, there is good empirical evidence that copulation alters numerous biological aspects of queen reproduction. These now well-characterized post-mating changes are important and quantifiable proxies that can be used for testing the effects of different copulation factors on queen post-mating changes.

## 3. Copulation Factors Influencing Queen Post-Mating Changes and Reproduction

Methods for instrumentally inseminating honey bee queens were developed in the early 1900s [98] and have been essential in understanding how specific aspects of mating affect queen quality and post-mating changes [98]. Queens that are five to seven days old are used for instrumental insemination. During insemination, they are anesthetized via exposure to a constant stream of CO_2_ [98]. Semen is expelled into the queen’s median oviduct using a glass microsyringe [98]. Studies involving the use of instrumental insemination have been used to disentangle how factors such as queen sexual receptivity, pheromone composition, and worker retinue response are affected by several copulation and instrumental insemination factors: CO_2_ exposure, physical manipulation of the vaginal tract, insemination volume, and insemination fluid composition (Table 1). It is important to note that exposure to CO_2_ alone or in combination with the physical manipulation that takes place during insemination can induce ovary activation and cessation of mating flight attempts (Table 1) [93,99]. Therefore, it is important to keep this in mind, especially when drawing conclusions in future research.

### 3.1. Effects of Drone Number and Insemination Volume on Post-Mating Changes

One of the earliest studies to suggest that insemination volume and/or drone mating number may contribute to differences in post-mating changes studied queens inseminated with semen from different number of drones [48]. Mandibular gland extracts [47] and Dufour’s gland extracts [48] from queens inseminated with semen from one drone (Single Drone Inseminated or SDI) (~1 μL) are less effective at stimulating worker retinue response than extracts from queens inseminated with semen from multiple drones (10 drones, ~10 μL) (Table 1). Furthermore, SDI queens are not as efficient as naturally mated queens in inhibiting worker ovary activation, likely due to differential modulation of mandibular gland pheromones [52]. On that same note, colonies headed by multiple-drone inseminated queens exhibit greater worker retinue response, build more comb, collect more pollen, store more honey, produce more brood, rear more drones, and exhibit higher rates of overwintering survival as compared to colonies headed by single-drone inseminated queens [47,100]. Furthermore, colonies headed by multiple-drone inseminated queens exhibit greater pathogen resistance [101,102,103].

Other studies specifically examined the effect of insemination volume on queen fertility and controlled for genetic diversity by inseminating all queens with semen from a single semen source pooled from a large number of drones. Queens inseminated with 8 μL of semen or saline solution tend to exhibit decreased sexual receptivity and increased ovary activation as compared to queens inseminated with 1 μL of semen or saline and virgins (Table 1) [58]. Furthermore, colonies headed by queens inseminated with lower volume tend to exhibit higher queen cell building/queen supersedure rates [50]. Increased insemination volume (8 μL versus 1 μL) also results in greater retinue response towards mandibular gland extracts [50,51], regardless of whether queens were inseminated with semen or saline (Table 1). In contrast, volume does not seem to effect Dufour’s gland extract composition, suggesting that activation of stretch receptors in the genital tract regulate mandibular gland secretions, but not Dufour’s gland secretions (Table 1) [51]. There are possibly stretch receptors in the queen’s median or lateral oviducts that expand during insemination and that are differentially activated based on insemination volume [51], similar to a mechanism found in moths; mechanical stimulation of the genital tract in the females of several moth species results in reduced production of sex pheromone [104].

At the worker and colony scale, Dufour’s glands of workers in colonies headed by queens inseminated with 8 μL of semen exhibited decreased proportions of esters, suggesting greater inhibition of worker ovary activation via QMP [50]. Intriguingly, colonies headed by queens inseminated with the higher volume exhibited higher overwintering mortality suggesting a potential trade-off between high pheromone production and queen/colony health [50], something that warrants further investigation.

### 3.2. Effects of Insemination Fluid Composition on Post-Mating Changes

In order to tease apart the role of components within semen in post-mating changes, saline serves as a good control when administered at the same volume. Most queens inseminated with semen or saline cease mating flights while virgin queens and queens that had recently accomplished one mating flight tend to remain sexually receptive [68]. While mandibular gland extracts of queens inseminated with 1 μL or 8 μL semen elicit a greater retinue response as compared to queens inseminated with saline solution of the same respective volumes, different insemination fluid composition does not affect Dufour’s gland extract composition [51]. This indicates that semen has components (e.g., proteins or metabolites) that initiate post-mating changes in queen mandibular gland secretions but not Dufour’s gland secretions [51].

Insemination fluid composition (saline versus semen) also differentially affects brain and ovary gene expression and has a greater impact on ovary gene expression than insemination volume, as queens inseminated with either 1 μL or 8 μL of semen had more similar ovary expression profiles than queens inseminated with saline of either volume [68]. However, insemination fluid composition does not affect brain gene expression, since queens instrumentally inseminated with semen or saline have expression profiles that are most similar to each other two days post-insemination and both exhibit an intermediate expression state between naturally-mated and virgin queens [68].

A recent study examined the role of seminal fluid, a component of semen, on queen visual perception using queens that were sedated on ice lieu of using potentially confounding CO_2_ exposure (Liberti et al. Baer, in review [105]). Queens inseminated with seminal fluid exhibit reduced brain expression of genes involved in phototransduction, similar to naturally mated queens (Liberti et al. Baer, in review [105]). They also start and finish mating flight attempts before control queens inseminated with buffer (Liberti et al. Baer, in review [105]). Further, preliminary data also suggests that drone seminal fluid, when injected in the queen abdominal cavity, reduces sexual receptivity and affects QMP production; queens injected with seminal fluid make fewer attempts at mating flights and tend to provoke higher worker retinue response compared to buffer-injected controls (Personal Communication with Elina L. Niño [106]).

The aforementioned studies indicate that the processes involved in queen post-mating changes are complex and are differentially affected by numerous factors such as insemination volume and insemination fluid composition. Honey bee seminal fluid contains proteins that likely serve as key drivers of seminal fluid-dependent post-mating changes in queens. Substantial work in *Drosophila* and other insects has determined that female post-reproductive changes can be largely attributed to the receipt of seminal fluid proteins rather than other mating components [107,108].

## 4. Seminal Fluid Proteins and Their Potential Roles in Queen Post-Mating Changes and Health

### 4.1. Seminal Fluid Functions in Drosophila and Other Insects

Male insect semen is composed of both spermatozoa cells and seminal fluid components [109,110,111,112,113,114]. Seminal fluid is a complex mixture of proteins and other small molecules, including peptides, sugars, and lipids, and is primarily derived from the male accessory glands, but other structures such as the ejaculatory bulb may contribute [110,115]. Mating results in fundamental changes in *Drosophila* females, where mated females exhibit shorter lifespans, lower metabolism, decreased receptivity to mating, increased oviposition rates, increased expression of immune-related genes, and overall differential gene expression compared to virgin flies [107,115,116,117,118,119,120,121,122,123,124,125]. These post-mating changes are caused, in large part, by the receipt of male accessory gland-derived seminal proteins during mating [126]. The influence of seminal fluid and seminal fluid proteins (SFPs) on male and female fertility and behavior, spermatozoa viability, and susceptibility to infection has been intensively studied for decades in *Drosophila* and increasingly studied and supported in other insects, including mosquitos, crickets, ants, moths, and beetles [14,107,108,115,116,117,119,120,121,122,123,124,125,126,127,128,129,130,131,132,133,134,135,136,137,138,139,140,141]. Insect SFPs typically encompass several different functional classes including proteases, protease inhibitors, lectins, coagulants, cysteine-rich secretory proteins, antioxidants, and antimicrobial proteins [107], which indicates conserved seminal fluid protein function across different insect species. However, SFPs vary highly at the primary sequence level and/or relative protein abundance and molecular mass, even when comparing sub-species or strains [107,142,143]. Research on *Drosophila* has identified specific proteins that reduce female sexual receptivity (i.e., sex peptide), maintain spermatozoa viability (i.e., Acp29AB), promote uterine contractions (i.e., Acp36DE), and ovulation (i.e., ovulin [144]). Interestingly, homologs of these specific proteins have not been identified within the honey bee genome, underscoring the uniqueness of the honey bee mating system and the need for specific investigation in this system. 

### 4.2. Identification of Honey Bee Seminal Fluid Proteins and Their Potential Roles in Queen Post-Mating Changes

To date, mass spectrometry has been used to identify the proteins in honey bee drone semen [111], accessory gland-associated proteins [111], seminal fluid [110,145,146,147,148], and spermatozoa cell-associated proteins [149]. Roughly 260 proteins have been identified in honey bee seminal fluid [148]. However, relative abundances and post-translational modifications of seminal fluid proteins differ between different genetic lineages of bees, which might at least be partially driven by sexual selection [145]. Male honey bees and other social hymenopteran insects are under strong selective pressures to produce high-quality ejaculates because queens only mate for a short period early in their lives and need to acquire sufficient numbers of spermatozoa to fertilize eggs for the rest of their reproductive lifespans [14,138,141,150,151,152]. Furthermore, seminal fluid influences spermatozoa competition, as the seminal fluid from one male can incapacitate the spermatozoa of other competing males [138].

Honey bee SFPs encompass several different biological pathways, including reactive oxidative species defense (e.g., superoxide dismutase 2) and metabolism (e.g., phosphoglycerate kinase), suggesting their roles in protecting spermatozoa against oxidative damage and supporting spermatozoa metabolism [110,148]. Indeed, whole seminal fluid maintains spermatozoa longevity [137,153]. While there is some research exploring potential functions of seminal fluid, their specific roles in initiating and maintaining queen post-mating changes have yet to be fully investigated. In regards to honey bee SFPs with potential effects on queen post-mating changes, drone seminal fluid also contains several odorant binding proteins and chemosensory protein 3 [110,148], which is present in bee antennae and binds to fatty acids in order to influence behavior [40]. Male bumblebees transfer fatty acids to queens, which results in reduced sexual receptivity [154,155,156]. Several odorant binding proteins also likely aid in solubilization and release of pheromones [157]. Thus, the transfer of chemosensory protein 3 and odorant binding/chemosensing proteins from honey bee drone seminal fluid into queens may induce chemical changes in the brain that influence queen cessation of mating flights and pheromone production/release [110]. However, this has not been tested empirically.

### 4.3. Roles of Honey Bee Seminal Fluid Proteins in Pathogen Defense

Several immune-related proteins are also found in honey bee seminal fluid, including chitinases, Osiris 7, and heat shock proteins, which have been associated with immune defense against pathogens [158,159,160,161]. Correspondingly, recent studies have determined that seminal fluid markedly reduces spore viability of the fungal pathogen *Nosema apis* [147,148]. Both protein and non-protein fractions of seminal fluid are able to reduce *N. apis* spore viability via two respective mechanisms [147]. Seminal fluid proteins induce germination-like rupture of the *Nosema* spore walls, whereas the non-protein fraction of seminal fluid directly decreases spore viability without cell wall rupture or spore germination [147]. When seminal fluid proteins are further separated into five fractions using solid phase extraction, three protein fractions exhibit antimicrobial activity against *N. apis* spores, which indicates there are multiple seminal fluid proteins with antifungal activity [147]. Components in the non-protein fraction have not yet been characterized, but they may be interesting research targets as potential mediators of queen post-mating changes.

It remains to be studied whether drone seminal fluid proteins also exhibit antibacterial or antiviral activity, but the honey bee seminal fluid proteome includes several heat shock proteins, which are important for antiviral immunity in *Drosophila* [162] and likely honey bees [158,159,161]. Queens mate with multiple males and therefore have an increased risk of acquiring pathogens through ejaculates [163]. Although they are ostensibly more resistant to pathogen infections as compared to workers [164,165], several pathogens, including viruses and *Nosema* spp., have been detected in queens (reviewed [28]). Viruses such as Deformed Wing virus can be transmitted to queens via instrumental insemination with contaminated semen [60] and queens from healthy colonies taking mating flights in areas containing colonies with high *V. destructor* mite infestation are more likely to be infected with Deformed Wing virus than queens located in low-mite infested areas [61]. In addition, Deformed Wing virus and Acute Bee Paralysis Virus have been detected in drone semen and endophalli [62,166,167]. Drones exhibiting high levels of Deformed Wing virus, >10^6^ genome copies per endophallus, have been detected in drone congregation areas (DCAs), so even highly infected drones are physically able to travel to DCAs and potentially mate and infect queens [167]. In contrast, drones parasitized as pupae by *V. destructor* mites, a major vector of Deformed Wing virus, exhibit decreased flight ability and spermatozoa counts [168]. Most notably, spores of the widespread fungal pathogens *N. apis* and *Nosema ceranae* have also been detected in honey bee semen [59]. They are able to infect queens if transferred during mating [59,169], even though they typically are transmitted via the fecal-oral route [170]. Drones infected with *N. ceranae* exhibit altered flight patterns but are able to maintain spermatozoa viability [171]. 

Drones infected by *N. apis* exhibit differential expression of 111 seminal fluid proteins, a large proportion of which are involved in immunity and detoxification [148]. The antifungal activity of seminal fluid collected from infected males is comparable to seminal fluid from healthy drones, except in diluted samples, for which seminal fluid derived from infected drones has reduced antimicrobial activity [148]. As *Nosema* infection alters pheromone production in queens [172], it would be relevant to address if semen of drones infected with *N. apis* also causes differences in other reproductive changes in queens. In addition, since viruses and *V. destructor* are prevalent throughout colonies and infect/infest drones, it will be important to determine how they alter seminal fluid composition and subsequent queen reproductive changes and health. 

There is now solid evidence that seminal fluid proteins are key drivers of post-reproductive changes in many species, and it is likely that honey bee drone seminal fluid proteins also influence post-mating changes in queens [110,148]. Furthermore, proteomic studies suggest that many of the proteins present in drone seminal fluid play key roles in spermatozoa maintenance, regulation of queen pheromone production and behavior, and antimicrobial defense [110,137,147,148]. Of these roles, the antimicrobial nature of drone seminal fluid/proteins has been the best studied thus far.

## 5. Conclusions and Future Directions

The queen is an important member of the honey bee colony and can be a major determinant of colony health and productivity. Drones, too, are very important players as they can have a strong impact on queen post-mating changes and subsequent colony health. Specifically, drone seminal fluid modulates queen sexual receptivity, pheromone production and seminal fluid proteins are likely the key drivers of these changes [105,106]. In order to further solidify the role of SFPs in queen post-reproductive changes, additional studies involving the separation of proteins from the non-protein fraction of seminal fluid and testing their effects on queen post-mating changes are forthcoming. Furthermore, specific SFPs and their functions could be identified via fractionation of proteins (e.g., ion chromatography) and testing their individual effects [147]. RNAi mediated gene knockdown [173] or CRISPR-Cas9 gene knockout [174] of genes encoding SFPs in drones will also likely provide exciting and enlightening paths towards a more holistic understanding of the functions of drone SFPs.

In addition, drone seminal fluid proteomes vary based on genetic lineage [145]. Based on these differences, future investigations should seek to understand if protein-level differences in honey bee seminal fluid composition due to genetic background (e.g., European versus Africanized bees) result in differential queen post-mating changes and if queens from different genetic lineages exhibit differential post-mating changes.

Furthermore, it is important to reiterate that honey bee seminal fluid is composed of both proteins and unidentified non-protein components, which are likely peptides, lipids, and sugars [110,115]. In addition to having antimicrobial activity against *N. apis* [147], the non-protein fraction of seminal fluid may also impact queen post-mating changes and reproduction. In two cricket species, *Teleogryllus commodus* and *Acheta domesticus*, prostaglandins present in their seminal fluid are responsible for reducing sexual receptivity and inducing oviposition in recipient females [175,176]. Prostaglandins are important for honey bee immunity [175], but it is unknown if they are also present in drone seminal fluid. Thus, more comprehensive studies, including metabolomics or peptidomics approaches, should yield insights into the role of non-protein fractions of seminal fluid in queen health and reproduction.

Lastly, identifying the specific roles of SFPs in queen reproduction could have an important impact on improving bee breeding practices in order to develop more resilient genetic honey bee stock. For example, being able to manipulate the production of specific SFPs in drones could lead to improved queen reproductive fitness particularly in breeder queens. Such selective breeding practices were utilized to develop *V. destructor*-resistant honey bees that exhibit higher expression of proteins associated with *V. destructor* resistance [177,178]. Ultimately, improving and understanding the underlying mechanisms of, and improving drone reproductive health has a great potential to improve resultant queen and colony health and contribute towards reducing colony losses.

## Figures and Tables

**Table 1:** Effects of different copulation factors on queen post-mating changes. Shown are outcomes of different treatments on queen post-mating changes, worker behavior, and colony longevity. Carbon dioxide (CO_2_) indicates queens that were exposed to CO_2_ alone but were not instrumentally inseminated. Virgins are control queens that were handled similarly to the other treatments, but were not inseminated or treated with any substance. CPM indicates queens that were both exposed to CO_2_ and physically manipulated in the oviduct to simulate the physical aspects of instrumental insemination. SDI are queens that were inseminated with semen from one drone (~1 μL) and MDI are queens that were inseminated with semen from multiple drones (~10 μL). The outcome of inseminating queens with 8 μL versus 1 μL semen is also shown. Saline serves as an insemination and volume control for semen in order to test the effects of semen components on post-mating changes. Lastly, seminal fluid is a component of semen that contains proteins (SFPs) that are likely important for inducing post-mating changes and Hayes solution is often used as a semen and seminal fluid diluent. “Yes, ns” in the table indicates that results trended toward the respective phenotype, but they were not statistically significant.

Mating/Insemination Factors								
Queen Post-Mating Outcomes	CO_2_ vs. Virgins	CPM vs. Virgins	SDI and MDI vs. Virgins	SDI vs. MDI	Insemination Volume: 8 μL vs. 1 μL	Semen vs. Saline	Seminal Fluid vs. Hayes	Naturally Mated vs. Virgin
**Reduced sexual receptivity?**	Yes [99]	Yes [93]	unknown	unknown	Yes, ns [58]	Yes, ns [55]	Yes [93]	Yes [13,63,68]
**Greater ovary activation?**	Yes, ns [93]; Yes [96,99]	Yes [93]	unknown	unknown	Yes [58]	Yes [55]	unknown	Yes [72,73,96]
**Enhanced worker retinue response?**	Yes [93]	No [93]	Yes [47,48]	Yes [47,48]	Yes [50,51]	Yes [51] Yes, ns [49]	Yes [93]	Yes [47,49,67]
**Modulated Mandibular gland pheromone production?**	Yes [93]	Yes [93]	Yes [47]	Yes [47]	Yes [51]	Yes [51]	unknown	Yes [49,54,55,84]
**Modulated Dufour’s gland pheromone production?**	No [93]	No [93]	Yes [48]	Yes [48]	No Difference [51]	No Difference [51]	unknown	Yes [65]
**# genes differentially expressed in brain out of all transcripts that were detected**	234/9091 [93]	504/9091 [93]	unknown	unknown	unknown	44/9850 [68]	unknown	576/10,468 [57] 180/9850 [68]
**# genes differentially expressed in ovaries out of all transcripts that were detected**	unknown	unknown	unknown	unknown	unknown	unknown	unknown	217/7377 [57] regulation of biogenic amine receptor genes [96]
